# Operative versus non-operative treatment in complex proximal humeral fractures: a meta-analysis of randomized controlled trials

**DOI:** 10.1186/s40064-015-1522-5

**Published:** 2015-11-25

**Authors:** Lin Xie, Fan Ding, Zhigang Zhao, Yan Chen, Danmou Xing

**Affiliations:** Department of Orthopedic Surgery, Wuhan Orthopedic Hospital, Wuhan Puai Hospital, Huazhong University of Science and Technology, Hanzheng Street 473#, Wuhan, 430033 Hubei China

**Keywords:** Operative, Non-operative treatment, Complex proximal humeral fractures (CPHFs), Meta-analysis, Randomized controlled trials (RCTs)

## Abstract

Whether operative treatment for complex proximal humeral fractures (CPHFs) has a greater benefit over non-operative treatment remains controversial. There is no consensus on the optimal treatment in elderly patients with CPHFs. This updated meta-analysis of randomized controlled trials (RCTs) aims to investigate whether operative treatment is superior to non-operative treatment in CPHFs. The authors searched RCTs in the electronic databases (Cochrane Central Register of Controlled Trials, PubMed, EMBASE, Medline, Embase, Springer Link, Web of Knowledge, OVID and Google Scholar) from their establishment to July 2015. Researches on operative and non-operative treatment for CPHFs were selected in this meta-analysis. The quality of all studies was assessed and effective data was pooled for this meta-analysis. Outcome measurements were functional status include constant scores (CS scores) and disabilities of the arm, shoulder and hand scores (DASH scores), total complication rates and healthy-related quality of life. The meta-analysis was performed with software revman 5.3. Nine articles with a total 518 patients (average age 70.93) met inclusion criteria. Patients were followed up for at least 1 year in all the studies. No statistical differences were found between operative and non-operative treatment in CS scores at 12 mo (months) [MD 1.06 95 % CI (−3.51, 5.62)] and 24 mo [MD −0.61 95 % CI (−5.87, 4.65)]. There are also no statistical differences between operative and non-operative treatment in DASH scores at 12 mo [MD −4.51 95 % CI (−13.49, 4.47)] and 24 mo [MD −7.43 95 % CI (−16.14, 1.27)]. Statistical differences were found between operative and non-operative treatment in total complication rates [RR 1.55, 95 % CI (1.24, 1.94)]. Statistical differences in EQ-5D at 24 mo [MD 0.15, 95 % CI (0.05, 0.24)] were found between operative and non-operative treatment but no statistical differences were found in ED-5D at 12 mo [MD 0.08, 95 % CI (−0.01, 0.17)], 15D at 12 mo [MD 0.02, 95 % CI (−0.68, 0.73)] and 15D at 24 mo [MD 0.02, 95 % CI (−0.07, 0.83)]. Operative treatments did not significantly improve the functional outcome and healthy-related quality of life in elderly patients. Instead, Operative treatment for CPHFs led to higher incidence of postoperative complications.

## Background

Proximal humeral fractures are common injuries that comprises 5–6 % of all adult fractures, with the incidence of 63.0/10^5^ per year (Bengner et al. 1988; Baron et al. 1996a, b). It is the third most common fracture after hip and wrist fractures that occur in patients older than 60 years (Roux et al. 2012; Horak and Nilsson 1975; Kannus et al. 1996). The fractures are common in patients older than 60 years especially females. Nearly 85 % proximal humeral fractures are non- or minimally displaced and can be treated conservatively (Roux et al. 2012). Many patients could regain shoulder function with non-operative treatment (Yuksel et al. 2011). The remaining 15 % displaced fractures which are challenge to surgeons can be treated with operative or non-operative treatment (Kim et al. 2012; Handoll and Ollivere 2010). These fractures include 2-part fractures involving the surgical neck, 3- and 4-part fractures which all have poor outcomes and the optimal treatment is still controversial. With recent advancement in technique and implants for fracture fixation (Russo et al. 2013; Lill et al. 2013; Vundelinckx et al. 2012), operative treatment has become increasingly popular for these injuries (Karataglis et al. 2011), including internal fixation (Jost et al. 2013) and humeral head replacement (Cadet and Ahmad 2012), which increased treatment costs for this fracture. While non-operative treatment includes sling immobilization (Yuksel et al. 2011).

To date, meta-analysis comparing conservation with operative treatment for proximal humeral fractures have been available in recent years (Sun et al. 2015; Mao et al. 2014; Jia et al. 2014; Fu et al. 2014; Li et al. 2013). However, they did not improve evidence-based decision making because of lack of RCTs. Recently, several RCTs have investigated whether operative treatment may provide greater benefits than non-operative treatment (Rangan et al. 2015; Fjalestad and Hole 2014; Fjalestad et al. 2010, 2012; Boons et al. 2012; Olerud et al. 2011a, b; Zyto et al. 1997; Stableforth 1984). Whether operative treatment for CPHFs has a greater benefit over non-operative treatment remains controversial. This updated meta-analysis of RCTs aims to investigate whether operative treatment is superior to non-operative treatment in CPHFs.

## Methods

### Search strategy

The authors conducted a search of the Cochrane Central Register of Controlled Trials, PubMed, EMBASE, MEDLINE, Springer Link, Web of Knowledge, OVID and Google Scholar up to July 2015. No language restriction was made. The search strategy first used Mesh terms [“Shoulder fractures” (Mesh) OR “Proximal Humerus fractures” (Mesh) OR “Proximal Humeral Fractures” (Mesh)] and type of clinical trial (randomized controlled trial) and then a secondary free search was performed using multiple keywords (humer* and fractur* and random*) to ensure inclusion all possible studies. In addition, the reference lists of the included studies were checked for eligible studies.

### Eligibility criteria

Studies were considered acceptable according to the following criteria: (1) complex displaced proximal humeral fractures; (2) operative treatment vs non-operative treatment; (3) functional outcomes, complications or healthy-related quality of life were described; and (4) randomized controlled trial study design. Studies were excluded if they (1) were abstracts, letters, or meeting proceedings; (2) had repeated data; or (3) enrolled patients with multi-trauma or patients undergoing surgery for a revision, infection, or nonunion or malunion.

### Data extraction

The eligible studies were reviewed and all appropriate data were extracted by two authors (LX, YC) independently. The extracted data included general demographic characteristics, functional outcomes, complications and healthy-related quality of life.

### Study quality assessment

The risk of bias of each study was independently assessed by two authors (FD, ZGZ), in accordance with the Cochrane risk of bias tool, which defines nine aspects: (1) random sequence generation (selection bias); (2) allocation concealment (selection bias); (3) blinding of participants (performance bias); (4) blinding of treatment providers (performance bias); (5) blinding of outcome assessors (detection bias); (6) intention to treat (attrition bias); (7) selective reporting (reporting bias); (8) comparable study groups; and (9) other bias. A qualification of risk of bias, including low risk, unclear risk, or high risk, was provided. The final qualification for each study was determined by consensus among three authors (LX, YC, and DMX).

### Statistical analysis

Statistical analysis was performed with revman 5.3 software (Cochrane Collaboration, Oxford, UK) for outcome measures. The outcomes were function outcome (CS scores; DASH scores; ASES; OSS and SF-12), complications (total complications rates; the rate of additional surgery; infection; avascular necrosis; osteoarthritis; nerve injury; nonunion; impingement and re-displacement) and healthy-related quality of life (EQ-5D, 15D). Continuous variables and dichotomous data were analyzed with mean difference (MD) and relative risk (RR), both with 95 % confidence interval (CI), respectively. Statistical heterogeneity was assessed by the I^2^ statistics. Fixed-effects model was used when the heterogeneity was negligible (I^2^ < 50 %). Otherwise a random-effects model was adopted. To define sources of heterogeneity, subgroup analyses based on internal fixation and arthroplasty were defined during the analysis design phase. Publication bias was tested by funnel plots when possible. P < 0.05 was considered statistically significant.

## Results

### Study selection and characteristics

**Fig. 1:**
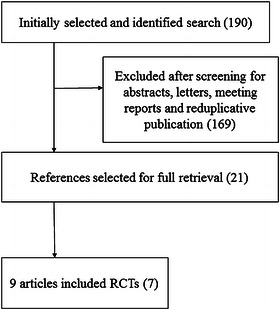
Flow diagram of literature search

Figure 1 illustrates the study flow. The initial search identified 190 references. After duplicate references were removed and the titles, abstracts, and contents of the full text were examined, 9 articles included 7 RCTs were included in the meta-analysis (Rangan et al. 2015; Fjalestad and Hole 2014; Fjalestad et al. 2010, 2012; Boons et al. 2012; Olerud et al. 2011a, b; Zyto et al. 1997; Stableforth 1984). Table 1 shows the general characteristics of the 9 included articles. A total of 518 patients (average age 70.93) with CPHFs were included in this study. Mean age ranged from 65.6 to 79.9. The percentage of female patients ranged from 75 to 96 %. The studies followed patients for periods of 12–60 mo, and the rate of patient follow-up ranged from 72.5–98 %.

**Table 1:** Characteristics of included studies (*O* operative, *N* non-opreative)

Study	Mean age (O/N)	No. (O/N)	% Female (O/N)	Follow-up (mo)	Rate of follow-up
Rangan et al. (2015)	67.42/66.12	114/117	77.6/76.0	24	86
Boons et al. (2012)	79.9/76.4	25/25	92/96	24	94
Fjalestad et al. (2010, 2012); Fjalestad and Hole (2014)	72.2/73.1	25/25	80/94	12	98.0
Olerud et al. (2011a, b)	72.9/74.9	30/30	80/83	24	88.3
Olerud et al. (2011a, b)	75.8/77.5	27/28	85/86	24	89.1
Stableforth (1984)	65.6/70.1	16/16	75/81.3	6–48	93.8
Zyto et al. (1997)	73/75	20/20	90/85	36–60	72.5

### Study quality

**Fig. 2:**
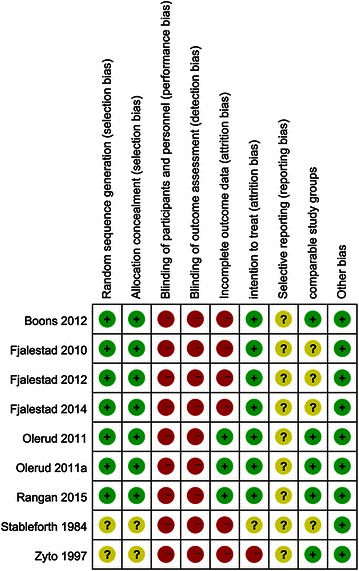
Risk of bias summary. Abbreviations: +, low risk; ?, unclear risk; −, high risk

Figure 2 shows the quality of the RCTs as independently assessed by two authors (LX, YC). Six studies were single-center studies and one study was multi-center study. five studies were judged as having used sufficient allocation concealment. Only one study used closed envelopes without reporting adequate safeguards. Only one study was reported to have blinded the outcome assessors. Six studies reported a proper intention-to-treat analysis and clearly stated interventions. The comparability of baseline characteristics was generally acceptable.

### Outcomes

#### Functional outcome

**Fig. 3:**
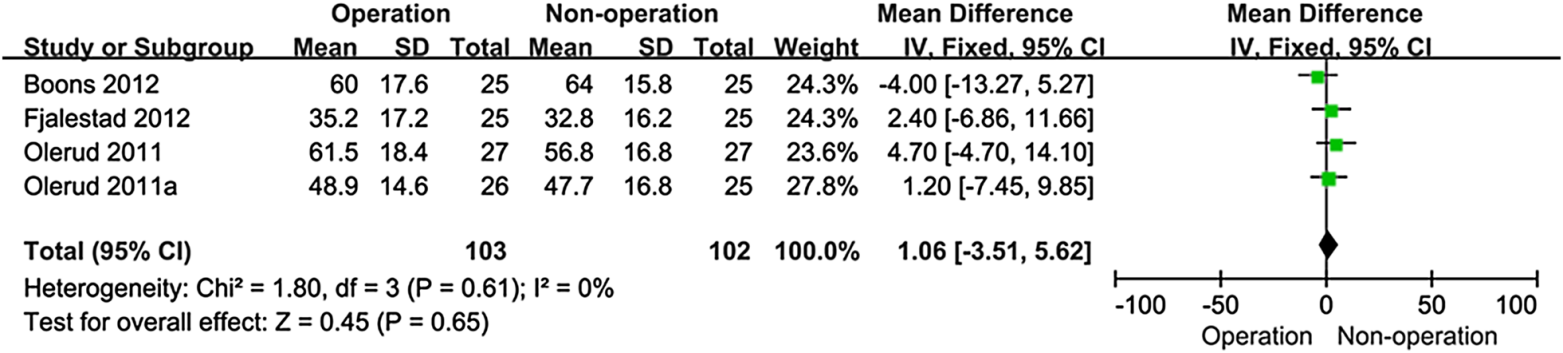
Forest plot for constant scores at 12 mo

**Fig. 4:**
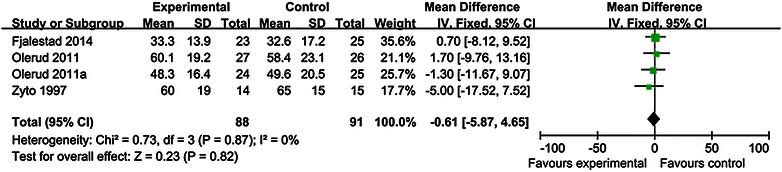
Forest plot for constant scores at 24 mo

**Fig. 5:**
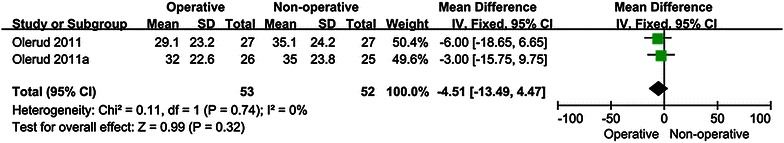
Forest plot for DASH scores at 12 mo

**Fig. 6:**
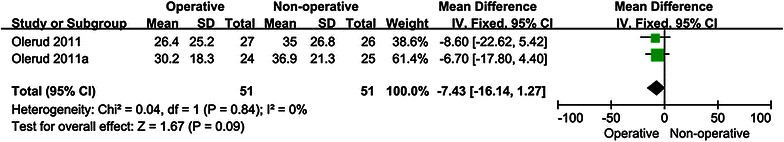
Forest plot for DASH scores at 24 mo

CS and DASH scores were the most commonly used to assess functional outcome of patients with displaced proximal humeral fractures. CS scores were mentioned in 6 studies and DASH scores were mentioned in two studies. No statistical differences were found between operative and non-operative treatment in CS scores at 12 mo [MD 1.06 95 % CI (−3.51, 5.62)] (Fig. 3) and 24 mo [MD −0.61 95 % CI (−5.87, 4.65)] (Fig. 4), There are no statistical differences between operative and non-operative treatment in DASH scores at 12 mo [MD −4.51 95 % CI (−13.49, 4.47)] (Fig. 5) and 24 mo (MD −7.43 95 % CI (−16.14, 1.27)] (Fig. 6), Other functional outcomes (ASES, OSS and SF-12) have no statistical differences between operative and non-operative treatment either (Table 2).

**Table 2:** Functional status outcome (*NA* not available)

Outcome	Studies	No. of patients (O/N)	MD (95 % CI)	P for MD	I^2^	P for heterogeneity
ASES score at 6 mo	1	23/25	0.10 (−3.66, 3.86)	0.96	NA	NA
ASES score at 12 mo	1	23/25	−0.70 (−4.52, 3.12)	0.72	NA	NA
OSS	1	114/117	0.75 (−1.45, 2.95)	0.50	NA	NA
SF-12 physical component score	1	111/115	2.00 (−1.00, 5.00)	0.19	NA	NA
SF-12 mental component score	1	111/115	−1.00 (−3.87, 1.87)	0.49	NA	NA

#### Complications

**Fig. 7:**
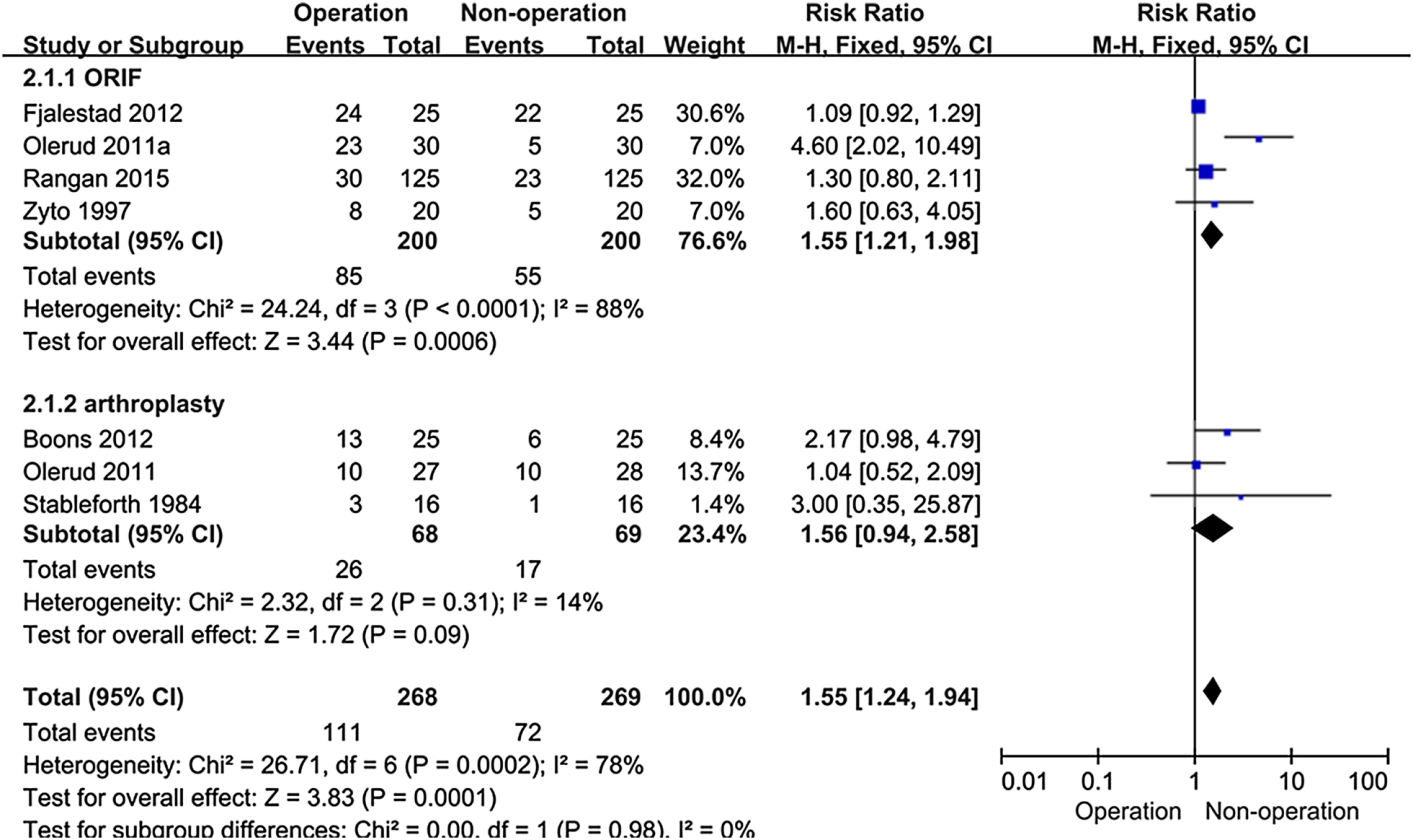
Forest plot for total complications rates

Total complications rates [RR 1.55, 95 % CI (1.24, 1.94)] have statistical differences between operative and non-operative treatment (Fig. 7). All the complication reported were summarized in Table 3. Six articles that included 497 patients provided data on the rate of additional surgery. The rate of additional surgery was significantly higher in the operative group compared with the non-operative group [RR 1.91, 95 % CI (1.06, 3.45); Table 3]. No statistical differences were seen in the rates of infection; avascular necrosis; osteoarthritis; nerve injury; nonunion; impingement or re-displacement between operative and non-operative treatment (Table 3).

**Table 3:** Total complication reported

Outcome	No. of trials	No. of patients (O/N)	RR (95 % CI)	P for RR	I^2^ (%)	P for heterogeneity
Additional surgery rate	Boons et al. (2012) Fjalestad et al. (2012) Olerud et al. (2011a, b) Rangan et al. (2015) Stableforth (1984)	248/249	1.91 (1.06, 3.45)	0.03	13	0.33
Mortality	Boons et al. (2012) Fjalestad et al. (2012) Rangan et al. (2015) Stableforth (1984)	191/191	2.07 (0.88, 4.83)	0.09	0	0.94
Infection	Fjalestad et al. (2012) Olerud et al. (2011a, b) Rangan et al. (2015) Zyto et al. (1997)	213/216	5.05 (0.90, 28.35)	0.07	0	1.00
Avascular necrosis	Fjalestad et al. (2012) Olerud et al. (2011a, b) Rangan et al. (2015) Zyto et al. (1997) Boons et al. (2012)	252/253	0.82 (0.38, 1.77)	0.61	16	0.31
Osteoarthritis	Fjalestad et al. (2010) Zyto et al. (1997)	41/41	1.34 (0.37, 4.82)	0.66	13	0.28
Nerve injury	Fjalestad et al. (2012) Rangan et al. (2015)	148/150	1.57 (0.65, 3.79)	0.32	0	0.38
Nonunion	Fjalestad et al. (2012) Olerud et al. (2011a, b) Rangan et al. (2015) Zyto et al. (1997)	189/191	0.38 (0.11, 1.26)	0.11	10	0.34
Impingement	Olerud et al. (2011a) Rangan et al. (2015)	149/150	1.02 (0.15, 7.05)	0.98	0	0.33
Redisplacement	Fjalestad et al. (2012) Olerud et al. (2011a) Zyto et al. (1997)	38/40	0.53 (0.10, 2.78)	0.45	48	0.16

#### Healthy-related quality of life

Only the differences in EQ-5D at 24 mo [MD 0.15, 95 % CI (0.05, 0.24)] were found between operative and non-operative treatment and no statistical differences were found in ED-5D at 12 mo [MD 0.08, 95 % CI (−0.01, 0.17)], 15D at 12 mo [MD 0.02, 95 % CI (−0.68, 0.73)] and 15D at 24 mo [MD 0.02, 95 % CI (−0.07, 0.83)] (Table 4).

**Table 4:** Health-related quality of life

Outcome	Study	No. of patients (O/N)	MD (95 % CI)	P for MD	I^2^	P for heterogeneity
EQ-5D at 12 mo	Olerud et al. (2011a, b)	53/52	0.08 (−0.01, 0.17)	0.10	0 %	0.83
EQ-5D at 24 mo	Olerud et al. (2011a, b)	51/51	0.15 (0.05, 0.24)	0.004	0 %	0.65
15D at 12 mo	Fjalestad et al. (2012)	23/25	0.02 (−0.03, 0.07)	0.44	NA	NA
15D at 24 mo	Fjalestad et al. (2012)	23/25	0.02 (−0.78, 0.83)	0.95	NA	NA

#### Sensitivity analysis

Due to the high heterogeneity in the above analysis, we performed subgroup analysis in the meta-analysis based on different surgical treatments. A sensitivity analysis was also conducted by removing one study at a time. We found that no article substantially influenced the results in this analysis.

#### Publication bias

The publication bias was evaluated by a funnel plot. The funnel plot shapes showed no obvious evidence of a symmetry. The results suggested that publication bias was not evident in this meta-analysis.

## Discussion

According to the Neer classification (Neer 1970a, b), the decision regarding the treatment of proximal humeral fractures is dependent on whether the four anatomical segments of the proximal humeral (the humeral head, shaft, and greater and lesser tubercles) are fractured or displaced. In our paper, CPHFs mean proximal humeral fractures excluding non- or minimally displaced proximal humeral fractures. CPHFs have poor outcomes and the optimal treatment is still controversial. There are several kinds of surgical methods for patients with CPHFs, including steosynthesis, hemiarthroplasty and reverse shoulder arthroplasty (Murray et al. 2011). Whether surgical methods could help to resume the painless range of motion and good shoulder function, thereby allow for rapid return to work and previous level of activity is still unclear. We performed this updated meta-analysis concerning the comparison of operative and non-operative treatment for the CPHFs. This meta-analysis was based on 7 RCTs in 9 articles (Rangan et al. 2015; Fjalestad and Hole 2014; Fjalestad et al. 2010, 2012; Boons et al. 2012; Olerud et al. 2011a, b; Zyto et al. 1997; Stableforth 1984). In this study, we compared the efficiency and safety of surgical and conservative interventions for CPHFs in elderly patients. The results of this meta-analysis indicated that surgical intervention only improved the ED-5D at 24 mo but suffered more complications. Meanwhile, no statistical differences were observed in CS scores, DASH scores, ED-5D at 12 mo and 15D.

Function outcome was a major clinical evaluation in all studies. Various measures have been developed to assess shoulder and arm disability. The measures can contain either self-reported or performance-based assessments or a combination of both. Among all these measure, the CS score is a widely accepted functional score of shoulder joint in the world (Constant and Murley 1987; Conboy et al. 1996; Rocourt et al. 2008). No statistical difference was detected with respect to CS score in our meta-analysis. The DASH score is a measurement of upper-extremity disability and symptoms (Hudak et al. 1996). There was also no statistical difference with regard to DASH score between two groups in our findings. Other functional outcomes (ASES, OSS and SF-12) have no statistical differences between operative and non-operative treatment either. Based on these outcomes, our analysis shows that operative treatment has no significant benefit on shoulder and arm functional recovery compared to non-operative treatment.

Total complications events in operative group were more common than that in conservative group especially the postoperative complications such as penetration of implant into joint rate, metalwork problem. For patients with operative treatment, the incidence of additional surgery is higher. This higher incidence of additional surgery in operative group may be associated with surgical expertise (Namdari et al. 2012). These complications could be minimized by improved operative techniques (Owsley and Gorczyca 2008; Egol et al. 2008). All deaths mentioned in this article were unrelated with interventions, thus, no statistically differences were found between operative and non-operative treatment in mortality. Some of these complication (infection; avascular necrosis; osteoarthritis; nerve injury; nonunion; impingement or re-displacement) may occur in both operative and non-operative treatment, and no statistical differences were found between operative and non-operative treatment in these complication.

Healthy-related quality of life in patients treated with operative treatment outperformed that with conservative treatments for EQ-5D at 24 mo, while no statistical differences in ED-5D at 12 mo, 15D at 12 mo and 15D at 24 mo (Table 4). While the number of studies included is limited. Only Olerud et al. (2011a, b) reported that hemiarthroplasty can improve EQ-5D at 24 mo, so more well designed, high quality RCTs are needed. All of these results indicate that neither operative nor non-operative treatment can achieve ideal clinical results, and operative treatment might fail to show a clinical benefit compared with non-operative treatment.

Although this meta-analysis was performed with the best available evidence presently, some unavoidable weaknesses earned to be noted. First, although we used multiple search strategies and available databases to include all possible studies, publication bias may be unavoidable. Second, the number of studies included is small. More well designed, high quality RCTs are needed. Furthermore, the types of operative or non-operative treatment in studies were varied and the follow-up periods in studies ranged largely from 1 year to several years. In addition, the variety of outcome measures limits the authors’ ability to combine outcomes and make definitive conclusions.

Although some limitations were unavoidable, this study has some merits. First, the search style based on the computer and manual search ensures a complete inclusion of relevant studies. Secondly, no significant heterogeneity was observed in most variables. Last, all the studies in this meta-analysis were RCTs.

## Conclusion

For CPHFs, current limited studies suggest that operative treatments did not significantly improve the functional outcome and healthy-related quality of life. Instead, Operative treatment for CPHFs led to higher incidence of postoperative complications. Based on the results of this meta-analysis, both treatment can achieve a similar effect on CPHFs, but operative treatment may increase the rate of total complication. Large, definitive RCTs are needed. Fortunately, such RCTs have already been designed (Launonen et al. 2012; Den Hartog et al. 2010; Handoll et al. 2009; Brorson et al. 2009).

## Abbreviations

CPHFs: Complex proximal humeral fractures
RCTs: Randomized controlled trials
CS: Constant
DASH: Disabilities of the arm, shoulder and hand
mo: Months
MD: Mean difference
RR: Relative risk
CI: Confidence interval
